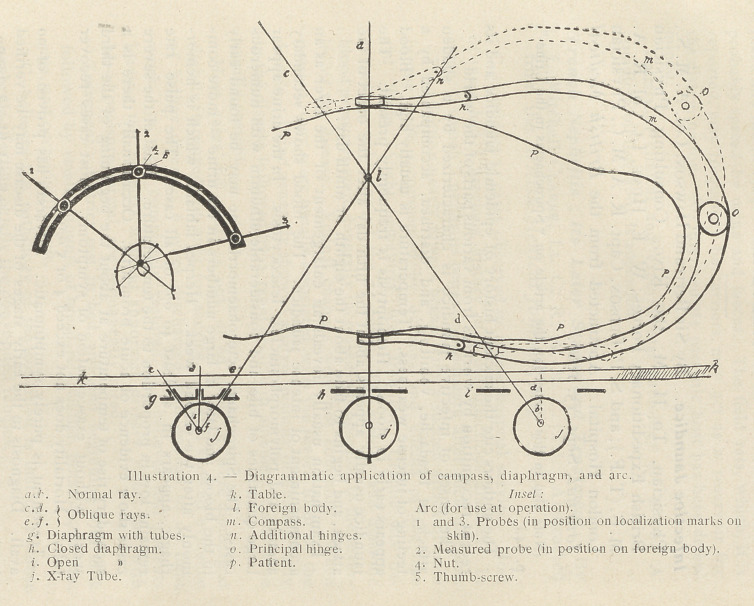# Simplified X-Ray Methods

**Published:** 1917-11

**Authors:** 


### RADIOLOGICAL

***Simplified X-Ray Methods.*** By HAROLD C. GAGE. Radiographer-in-chief of Hôpital Militaire N° 76. Consulting Radiographer and to the American Red Cross Hospital of Paris. Abstracted by the author from the *Archives of Radiology and Electrotherapy*, June 1917.

The following method is undoubtedly the best for general localization. It is independent of mathematical calculations, absolutely accurate, and the results are self-proving. The method is a screen one; the appliances necessary can be home-made.

Two or three pairs of compasses are shaped as in Illustration I, Figs. 1 and 2. Several sizes are necessary, as it is desirable that the rings shall be parallel when in use : a large pair (for the body) about 14 inches long; a second pair about 10 inches; a third pair about 5 inches. The rings in each case can be made to enclose smaller rings to facilitate the centering of a tiny foreign body. The patient is first placed, if a horizontal table be used, upon his back. Long sandbags are placed under the patient on either side of the area of localization to permit the insertion of the compasses beneath the limb or body. The tube having been previously centered, the diaphragm is closed down upon the foreign body. Observations in the antero-posterior position are made, adjusting the compasses in such a manner that the foreign body appears on the screen encircled by the rings. The skin is marked through those rings with a blue grease paint, and the patient then rotated. The foreign body is again encircled with the rings of the compass, and further skin markings made with a grease paint of another color. This marking is then repeated in another position, making three positions in all, and giving six marks of three colors upon the skin.

Reference to Illustration 1, Figs. 5, 5*a*, 5*b*, show strips of soft malleable metal made of an alloy used for high frequency electrodes. The strips should be of various lengths sufficient to encircle the different circumferences of limbs and body. They are hinged in the center. This metal band should now be moulded to the exact

Illustration 1. — Localizing Appliances.

contour of the body at the level of the localization marks. Where the metal overlaps, a line is drawn, and the colored markings on the skin are added to the metal band; also the wound of entrance or the incidence of the perpendicular drawn from it. The metal is then lifted (care being taken to see that the contour is preserved) and placed upon a sheet of paper. With a pencil its internal contour is transferred to the paper, as also are the colored marks.

The anatomical level of the foreign body should be noted, and, if the wound of entrance is not in the same place, its distance above or below measured. Until the worker is familiar with the method, large wooden callipers may be used to take the lateral antero-posterior measurements of the body, in order to confirm the shape and position of the transferred metal. The colored marks are connected with the aid of a ruler, and it will be found that the three lines intersect. (Should this not be the case it is obvious that some error in technique has been committed.) The intersection will represent the position of the foreign body. The grease paint marks upon the skin may be rendered permanent by nitrate of silver.

In the case of a very sick patient where it is impraticable to turn him, he may be placed on a small table at right angles to the X-Ray table. In this manner a long displacement of the tube is provided across the patient. The first antero-posterior observation is made as before, then the tube is displaced to the left and the foreign body encircled as before with the rings, and repeated with tube displaced to the right after which the proceedings are as before.

If it is desired to use the parallax method of localization and a cross section chart produced, the tube may be centred under the foreign body and the anterior and posterior marks placed upon the skin, after which a plate is placed over the anterior and an exposure made. The tube is then displaced at a known distance and a second exposure made. In this manner a wall diagram can be used for the purpose of calculating the depth of the foreign body on this vertical line, as the displacement will be in the same plane. The contour of the body at the level of the markings should now be taken and transferred to a diagram with the anterior-posterior markings; these are connected by a line, and the position of the foreign body recorded. Other marks may now be placed upon the outlined contour of the body in such a manner that lines drawn through them will intersect at the position of the foreign body; the marks are then transferred to the skin by replacing the malleable metal band. In this way choice of entrance with fixing points for the arc will be available at the operation, and the advantages of a " Cross Section Anatomy " utilised.

*The Chart and its Amplifications.*—Whichever method may have been chosen for the reproduction of the skin markings, the proceding for transferring them and the contour of the limb or body upon paper is the same, and the same intersecting lines are drawn. The cross section anatomical details of the area at the level of the foreign body may then be filled in. Reference to Illustration 2 will show in what manner these graphic amplifications may be made. Lines may be drawn showing the path of the projectile, and the chart will thus disclose not only the anatomical situation of the

Illustration 2.—The anatomical amplification of the chart.

foreign body, but also the route it has taken to reach its position, as well as any vessels or organs which may have been injured in its transit. In the event of the wound being somewhat remote, other cross section diagrams at intervals will be of considerable help, or, if a sagittal section of the area is available, work may be saved by reference to it. With a chart so constructed the surgeon has definite information as to the exact position of the foreign body, with full confidence in the absolute accuracy of its localization. He also knows the positions of organs or vessels of surgical importance, near or distant, and by a glance at this chart the easiest approach for removal of the foreign body is at once obvious. If it has been observed in the radiograph that a bone, not in the direct course from wound to projectile, has been injured, the path of the projectile would be from wound to bone injury and from bone injury to the localization. Transparent paper can, of course, be used in the preparation of these diagrams, so that, if desired, they can be superimposed on a " Cross Section Atlas " and the anatomical details traced in.

*Localization of Foreign Bodies in the Head.* — Additional precautions are necessary for the localization of a foreign body in the head. An exact localization can be made by using a length of wire to embrace the circumference of the head and passing it through the two marks of the first observation. It is then possible, during the subsequent observations, to turn the head in such manner as to maintain the same plane, and to adjust the former so that the wire intersects the foreign body as a line and does not appear on each side as an ellipse.

Further observations in localization of foreign bodies in the head can be made by using a strip of soft metal, as previously described, for transferring the contour of the part. The metal should be placed so as to take the contour of the dome of the head vertically over any pair of localization marks. The points chosen should be those best suited to the " Cross Section Anatomy " one may have at hand. It does not really matter which observation is taken so long as the two marks are on the same localization line passing through the foreign body. This contour can be added to the same localization line on the chart (see Illustration 3), and a line drawn from the foreign body either to some part of the vault of the skull through which a surgeon would desire to trephine, or to the wound

Illustration 3. — Localization chart of head showing contour of the dome added to the localization line.

of entrance. If the line does not intersect the wound, or the most practical point of entrance, an observation can be made for the purpose. It will thus be seen that by choosing either of the three lines, or a fourth line made specially, the foreign body may be reached definitely through any desirable point or previous opening. The scalp can be marked by replacing the metal band, and at the subsequent operation the arc described below may be adjusted for the surgeon.

*Mechanical guidance for use at the operation.* — This arc (see inset of Illustration 4) is made of metal, and is constructed to take three movable fittings, each being bored for the passage of a probe. These fittings, which are in the form of composite nuts (Fig. 4) can be firmly fixed in any position on the arc while the probes (Figs, 1, 2, 3) are still left perfectly free for movement, the latter being fixed when desired by a separate thumb screw (Fig. 5) fitted in the nut. One of the probes (Fig. 2) is marked in millimeters. The arc is placed upon the diagram, the measured probe being placed on the point corresponding to that chosen by the surgeon for his incision, and the others on any other two localization marks within reach. The nuts and screws of the two latter are now firmly fixed; the nut alone of the measured probe is made secure, and the probe is allowed to travel forwards to the position indicated as that of the foreign body. Notice is taken by the surgeon of the exact depth at which the point of this probe touches the foreign body on the chart through the area of the incision. The whole is then sterilized. This simple apparatus can be placed on the marks in the field of operation, and as the incision is made the central probe will mechanically follow, until, at the depth previously ascertained, it touches the foreign body.

Care should be taken to place the patient upon the operating table in the position he occupied when the markings were made. Although this is not so important as in most other methods of localization, it is obvious that a localization made in pronation would be invalidated should the operation be performed with the limb flexed or in supination. In other than gross changes like these the marks will be sufficient to give correct information.

*Bromide Paper Substituted for Plates.* — The author emphasizes the great value of bromide paper in war radiography : to quote —

" Bromide paper and the economy it offers, should not be forgotten, particularly in view of the comparatively large quantity one can transport. It is quite possible to make very good radiographs with rapid bromide paper if an intensification screen be used for deep parts and for the limbs. It has been found that to get the best results when using intensifying screens, it is necessary to have the equivalent spark-gap of the tube some 25 or 30 per cent

Illustration 4. — Diagrammatic application of campass, diaphragm, and arc.

*a.b.* Normal ray.
*c.d.* } Oblique rays.
*e.f.* }
*g.* Diaphragm with tubes.
*h.* Closed diaphragm.
*i.* Open "
*j.* X-ray Tube.
*k.* Table.
*l.* Foreign body.
*m.* Compass.
*n.* Additional hinges.
*o.* Principal hinge.
*p.* Patient.

*Inset:*

Arc (for use at operation).

1 and 3. Probes (in position on localization marks on skin).

2. Measured probe (in position on foreign body).

4. Nut.

5. Thumb-screw.

less than one would use for direct radiography. This also applies to the use of plates. *Bromide prints made in this way are particularly useful when only one copy is required to accompany an evacuation.*"

## Figures and Tables

**Illustration 1. f1:**
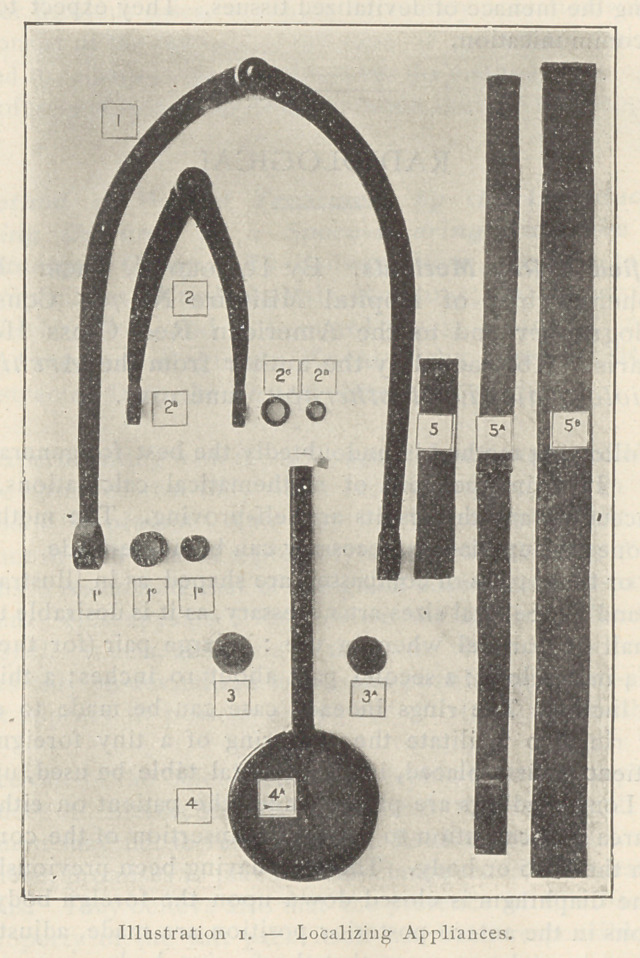


**Illustration 2. f2:**
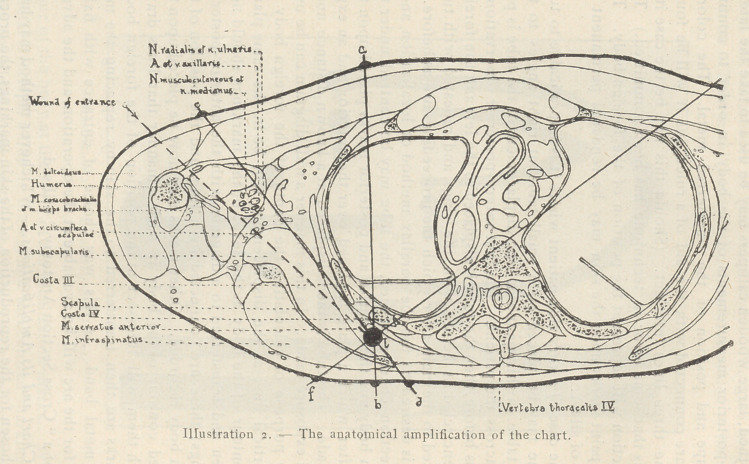


**Illustration 3. f3:**
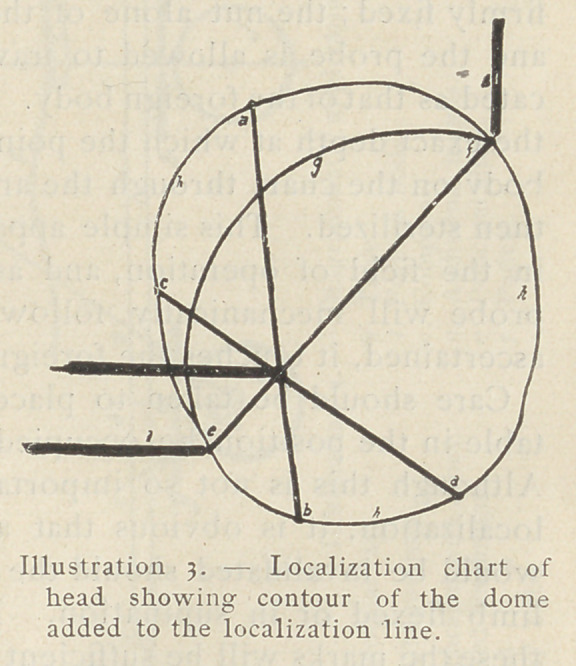


**Illustration 4. f4:**